# Functional and population genetic features of copy number variations in two dairy cattle populations

**DOI:** 10.1186/s12864-020-6496-1

**Published:** 2020-01-28

**Authors:** Young-Lim Lee, Mirte Bosse, Erik Mullaart, Martien A. M. Groenen, Roel F. Veerkamp, Aniek C. Bouwman

**Affiliations:** 1 Wageningen University & Research, Animal Breeding and Genomics, P.O. Box 338, Wageningen, AH 6700 the Netherlands; 2 CRV, Arnhem, the Netherlands

**Keywords:** Copy number variations, *Bos taurus*, Linkage disequilibrium, Population genetics

## Abstract

**Background:**

Copy Number Variations (CNVs) are gain or loss of DNA segments that are known to play a role in shaping a wide range of phenotypes. In this study, we used two dairy cattle populations, Holstein Friesian and Jersey, to discover CNVs using the Illumina BovineHD Genotyping BeadChip aligned to the ARS-UCD1.2 assembly. The discovered CNVs were investigated for their functional impact and their population genetics features.

**Results:**

We discovered 14,272 autosomal CNVs, which were aggregated into 1755 CNV regions (CNVR) from 451 animals. These CNVRs together cover 2.8% of the bovine autosomes. The assessment of the functional impact of CNVRs showed that rare CNVRs (MAF < 0.01) are more likely to overlap with genes, than common CNVRs (MAF ≥ 0.05). The Population differentiation index (Fst) based on CNVRs revealed multiple highly diverged CNVRs between the two breeds. Some of these CNVRs overlapped with candidate genes such as *MGAM* and *ADAMTS17* genes*,* which are related to starch digestion and body size, respectively. Lastly, linkage disequilibrium (LD) between CNVRs and BovineHD BeadChip SNPs was generally low, close to 0, although common deletions (MAF ≥ 0.05) showed slightly higher LD (*r*^*2*^ = ~ 0.1 at 10 kb distance) than the rest. Nevertheless, this LD is still lower than SNP-SNP LD (*r*^*2*^ = ~ 0.5 at 10 kb distance).

**Conclusions:**

Our analyses showed that CNVRs detected using BovineHD BeadChip arrays are likely to be functional. This finding indicates that CNVs can potentially disrupt the function of genes and thus might alter phenotypes. Also, the population differentiation index revealed two candidate genes, *MGAM* and *ADAMTS17*, which hint at adaptive evolution between the two populations. Lastly, low CNVR-SNP LD implies that genetic variation from CNVs might not be fully captured in routine animal genetic evaluation, which relies solely on SNP markers.

## Background

Genetic variations exist in various forms in genomes. Although single nucleotide polymorphisms (SNPs) have been the choice of variants in numerous studies, there is a growing body of evidence that copy number variations (CNVs) can have functional impact. Copy number variations are DNA segments of 1 kb or larger, and are present in varying copy numbers, compared to a reference genome [1]. Since the initial discovery of large sub-microscopic CNVs (some hundred kb) [2, 3], rapid developments in detection platforms and algorithms have advanced knowledge about CNVs, mainly in humans [4, 5].

In the early phase of their discovery, CNVs were expected to resolve the missing heritability (significant SNPs identified from genome-wide association studies (GWAS) together account small part of the heritability) [6, 7]. It was because, as in terms of base pairs, they cover a larger proportion of the genome, compared to SNPs. With the accumulation of data and analyses, the occurrence of CNVs in the genome was shown to be biased outside of functional elements [5]. Nevertheless, numerous studies have shown that CNVs play a role in determining a wide range of human health conditions, from obesity to neurodevelopmental diseases [8–11]. For instance, high copy numbers of the *CCL3L1* and *CYP2D6* genes confer reduced susceptibility to infection with HIV and the development of AIDS [12]. Also, the role of CNVs in adaptive evolution is further exemplified by mean copy numbers of the *AMY1* gene (which codes for amylase alpha1, an essential enzyme for starch digestion). The mean copy number of *AMY1* gene was shown to differ in human populations depending on dietary starch composition [13]. These findings demonstrate that CNVs may contribute to adaptive potential, and thus contain information about population history.

Studies in livestock species also highlighted the role of CNVs in shaping various phenotypes. For example, several genes affected by CNVs determine coat colours of specific breeds. Duplications of the *KIT* gene in pigs are related to white coat, which is only shown in domestic pigs [14, 15]. In cattle, serial translocation of the *KIT* gene was related to a colour-sidedness phenotype [16]. Moreover, CNVs were shown to be associated with quantitative traits that are economically important in livestock breeding, in various cattle populations [17–19]. One study investigated whether trait associated CNVs are in linkage disequilibrium (LD) with, and thus are tagged by, SNP markers, and revealed that ~ 25% of CNVs were not in LD with SNP markers [17]. However, this study was based on Illumina BovineSNP50 array data, in which SNP density and CNV resolution were low.

Holstein Friesian (HOL) and Jersey (JER) are the two main commercial dairy cattle breeds that have been bred under different breeding schemes. Although there have been studies investigating the link between CNVs and individual production traits [17–21], in-depth assessment of functional impacts of CNVs in cattle genomes has been limited. Also, whether CNVs that have an impact on phenotypes are captured in genomic evaluation, in other words, whether CNVs are in sufficient LD with SNPs, is largely unexplored. Furthermore, CNVs have been shown to be useful in disentangling population history and provide valuable insights in understanding how populations have evolved over time [22–25]. However, population genetics analyses exploring CNVs, with their main focus on HOL and JER, have been sparse.

Here, we aimed at discovering CNVs in bovine genomes based on genome assembly ARS-UCD1.2 [26] using high density SNP array data, in two dairy cattle populations. Subsequently, we performed in-depth analyses on the functional impact of CNVs and further explored the population genetic features of CNVs by analysing population differentiation index (Fst) and LD.

## Results

### CNV discovery in the genome build ARS-UCD1.2

The data consisted of Illumina BovineHD BeadChip (Illumina, San Diego, CA, USA) genotypes from two distinct dairy breeds (Holstein Friesian – HOL (*n* = 331), Jersey – JER (*n* = 115)) and their crossbreds (*n* = 29). A previous study using PennCNV on BovineHD data, of which 47 HOL animals overlapped with our study, showed high rate of CNV confirmation based on qPCR validation (91.7% for CNVs found in multiple animals, 40% for singleton CNVs) [24]. Therefore, we chose to perform CNV detection on bovine autosomes using the PennCNV software [27]. The Bovine HD SNPs were aligned to genome assembly ARS-UCD1.2.

**Fig. 1:**
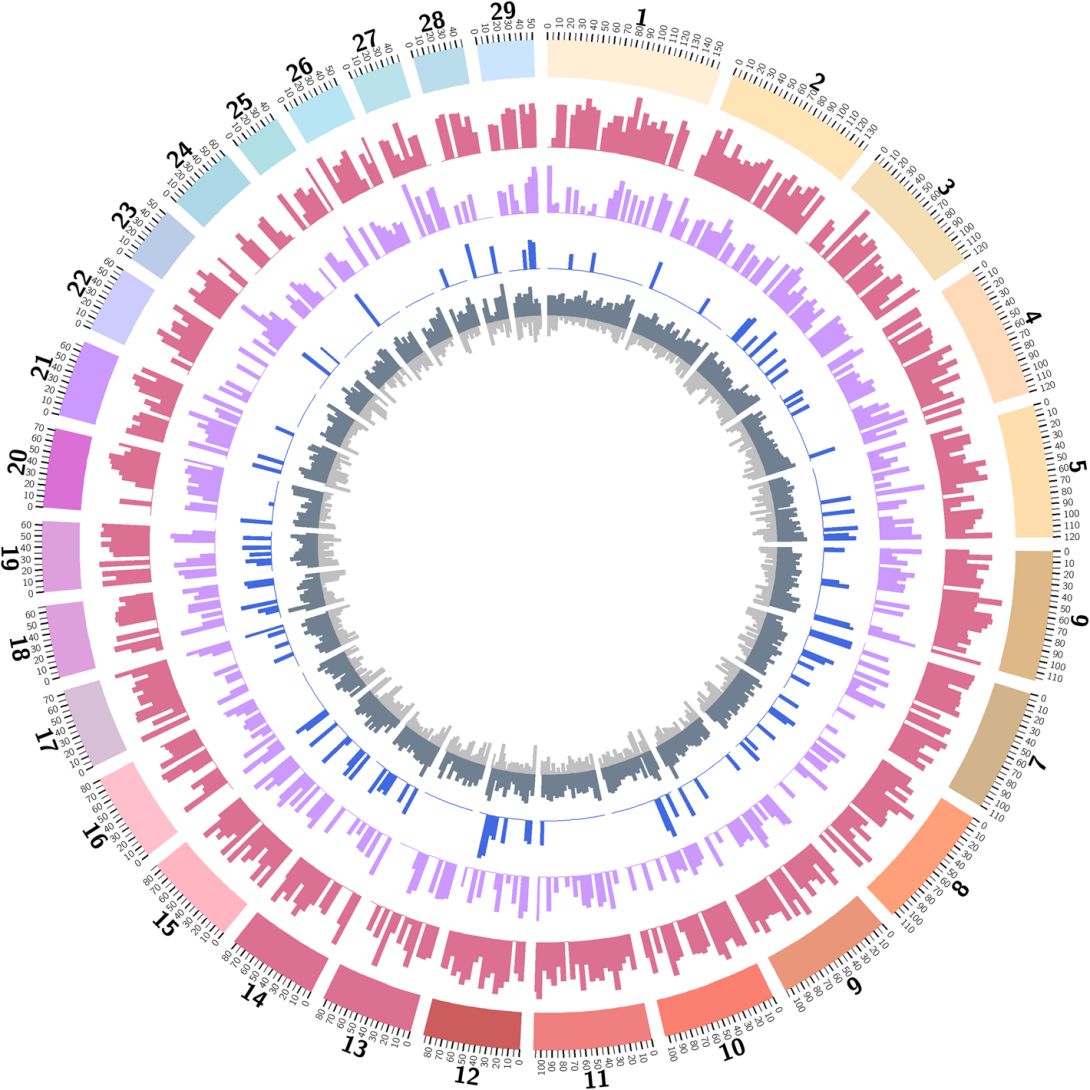
Circular map of autosomal copy number variant regions and their population genetics features. From the outside to the inside of the external circle: chromosome name; genomic location (in Mb); histogram representing density of deletion CNVRs in 5 Mb bin (pink); histogram representing density of duplication CNVRs in 5 Mb bin (purple); histogram representing density of complex CNVRs in 5 Mb bin (blue); number of BovineHD BeadChip array SNPs in 5 Mb bin (dark grey); histogram representing density of segmental duplications in 5 Mb bin (light grey)

We discovered 14,272 CNV calls from 451 individuals that passed the quality control criteria (31.6 calls/individual). Deletion calls were 1.8 times more frequent but 40% shorter (*n* = 9171, mean length = 44.2 kb) than duplication calls (*n* = 5101, mean length = 74.6 kb; Additional file 2: Table S1 and Additional file 1: Figure S1). The mean probe density (number of supporting SNPs per Mb CNV) was 403 SNPs/Mb. The 14,272 CNV calls were aggregated into 1755 CNV regions (CNVRs), based on at least 1 bp overlap, following Redon et al. [28]. These CNVRs cover 2.8% of the autosomal genome sequence (69.6/2489.4 Mb; Fig. 1; A full list of CNVR is in Additional file 2: Table S2.). These CNVRs consist of 1125 deletion CNVRs (mean length = 29.2 kb), 513 duplication CNVRs (mean length = 36.8 kb), and 117 complex CNVRs (mean length = 152.7 kb). The distribution of CNVR length is exponential, where the majority CNVRs are short to medium length (< 100 kb, 93%), while only a few observations are made for long CNVRs (> 100 kb, 7%). The CNVRs are non-randomly distributed over the chromosomes: chromosome-wide CNVR coverage varies from 0.6% on BTA24 to 4.9% on BTA12 (Additional file 2: Table S3). BTA12 is most densely covered with CNVR in terms of bp (4.2 Mb), and especially enriched for complex type CNVRs (2.2 Mb). Allele frequency of CNVRs ranges between 0.001 and 0.21.

Since most cattle CNV studies used genome assembly UMD3.1, we also repeated the CNV detection procedures, using UMD3.1. Subsequently, we used these calls to assess our CNV discovery results with other cattle CNV papers. From the 447 individuals that passed the QC criteria, 24,264 CNVs were called (54.3 calls/individual) and the mean probe density was 326 SNPs/Mb. These CNVs were aggregated into 1866 CNVRs (1130 deletions, 593 duplications, and 143 complex CNVRs). The mean length of deletion, duplication, and complex CNVRs is 29, 36, and 193 kb, respectively (Additional file 2: Table S1). These CNVRs together cover 82 Mb (3.3%) of bovine autosomes. The chromosome-wide coverage varies between 1% on BTA24 and 10% on BTA12 (Additional file 2: Table S4 and Additional file 1: Figure S2). Compared to other cattle CNV studies conducted using the same SNP array and the genome assembly UMD3.1 [22, 24, 29–32], our CNV discovery results are in a similar range (Additional file 2: Table S5).

When we compared to our CNVs discovered based on UMD3.1 and ARS-UCD1.2, we observed several differences. Firstly, the number of CNVs called per individual based on ARS-UCD1.2 is 42% lower than what was obtained using UMD3.1. Also, the mean probe density increased from 326 SNPs/Mb in UMD3.1 to 404 SNPs/Mb in ARS-UCD1.2, indicating that with ARS-UCD1.2, CNVs are supported by more SNPs. Lastly, the mean length of complex CNVRs decreased by 40 kb, from 193 kb in UMD3.1 to 152.7 kb in ARS-UCD1.2. We further inspected BTA12:70–77 MB region where a large change between UMD3.1 and ARS-UCD1.2 was observed. This region was reported to have a large number deletion and duplication calls by other cattle CNV studies based on UMD3.1, regardless of the studied breeds [24, 29–33]. In our CNV discovery, we identified 7 CNVRs (total length of ~ 6.2 Mb) in this region based on UMD3.1, whereas ARS-UCD1.2 based results revealed 9 CNVRs that covered ~ 1 Mb. We compared the positions of BovineHD SNPs in UMD3.1 and ARS-UCD1.2 to see whether the changes in genome assemblies caused this discrepancy. The results showed that 43% of the SNPs located in BTA12:70-77 Mb based on UMD3.1 were either moved to unmapped contigs or reference and alternative SNPs were undefined. The genome-wide ratio of SNPs that were moved to different chromosomes or contigs was much lower (2.3%) than 43%. This indeed indicates that the two genome assemblies differ in this regions, and thus led to different CNV discovery results.

### Functional impact of CNVRs

**Fig. 2:**
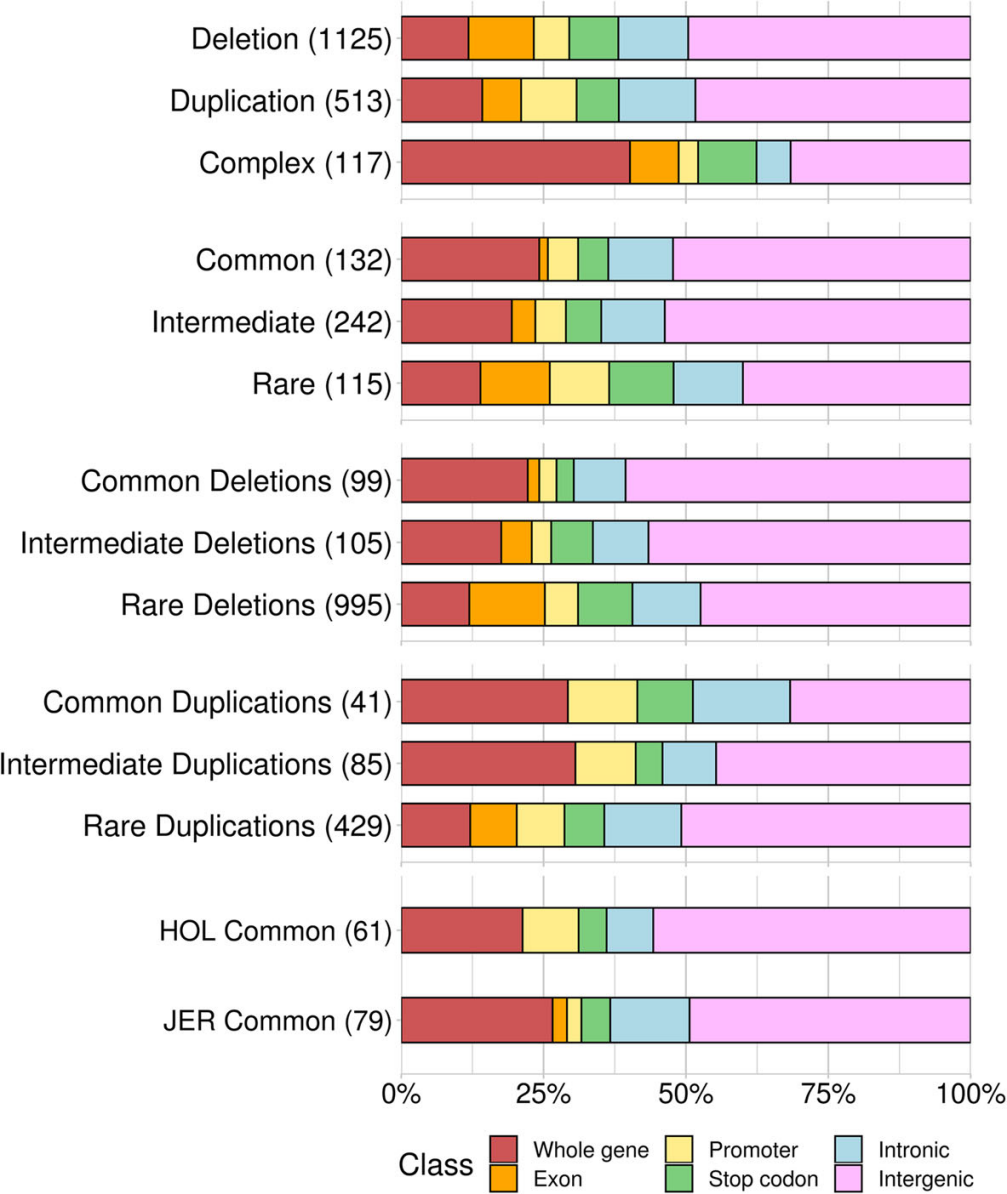
Functional impact of CNVRs by type, frequency, and population. Functional impact of CNVRs were investigated by type, frequency, and population. CNVRs were categorized into different types (deletion, duplication, and complex) and frequency (common: 0.05 ≤ MAF in any population, intermediate: 0.01 ≤ MAF < 0.05, rare: MAF < 0.01 in all populations). The numbers in the brackets indicate the number of CNVRs in each category

The expression of genes can be altered by CNVs. Deletions and duplications of a part of and/or complete gene can disrupt the gene expression and can potentially lead to changes in various phenotypes [34]. Therefore, identification CNVRs that coincide with genes can be a primary step to assess their functional impact. To achieve this, we explored CNVRs found based on ARS-UCD1.2 further. The overlap of CNVRs with Ensembl annotated genes were analysed, and among the 1755 CNVRs, 912 (52%) are genic and 843 (48%) are intergenic. Genic CNVRs overlap with 1739 genes out of 27,570 Ensembl annotated genes (6.3%) and 2936 out of 43,949 gene transcripts (6.7%). Among the 1739 genes that overlap with CNVRs, 957 (55%) are completely within the CNVRs and the rest (45%) are partially affected (genic features were inside the CNVRs). The following functional impact categories were assigned to each CNVR depending on types of overlap between CNVRs and genes (numbers in the brackets indicate number of CNVRs and genes respectively for each category; see materials and methods for detailed explanation for the classification): 1) intergenic (843 CNVRs; 0 genes), 2) intronic (214 CNVRs; 234 genes), 3) whole gene (253 CNVRs; 957 genes), 4) stop codon (147 CNVRs; 203 genes), 5) promoter regions (124 CNVRs; 187 genes), and 6) exonic (174 CNVRs; 165 genes). Then, these functional categories were intersected with other features of CNVRs such as types (deletion, duplication, complex), MAF (common, intermediate, and rare; see methods for detailed explanation), and the populations (HOL and JER; Fig. 2). The functional consequences of CNVRs differ depending on the type of CNVRs: Complex CNVRs were skewed towards genic regions (68% are genic), whereas deletions and duplication CNVRs were biased away from genic regions (51–52% are genic), and the difference is significant (chi-square test *P* < 10^− 13^). Also, we observed that MAF have impact on different types of overlap between genes and CNVRs. Rare CNVRs tend to be genic more often (60%), whereas common CNVRs have less overlap compared to it (48%; chi-square test *P* < 0.002). However, when seen it separately for deletion CNVRs and duplication CNVRs, we saw a different pattern. Common deletion CNVRs are more often intergenic (61%), yet the common duplication CNVRs are often genic (68%). When CNVRs between HOL and JER are compared, common JER CNVRs are more often genic (51%), than common HOL CNVRs (44%). Subsequently, we performed permutation tests on overlaps between CNVRs and autosomal genes, to test whether the overlap is significantly higher than expected under a neutral scenario. The results show that CNVRs overlap with autosomal genes more often than what is expected from permutation tests with random genomic regions (*P* < 0.001). Nextly, gene ontology analyses were performed to understand the functions of the genes that overlap with CNVRs. Genes overlapping deletions, duplications, and complex CNVRs were tested for GO enrichment as separate classes (Table 1). Among the findings, genes overlapping with the complex CNVRs (*n* = 407) show a pronounced enrichment in response to stimulus (GO:0050896; FDR = 1.8 X 10^− 6^), immune response (GO:0006955; FDR = 1.9 X 10^− 3^), and detection of stimulus involved in sensory perception (GO:0050906; FDR = 1.1 X 10^− 2^). These findings are similar to the findings from earlier cattle CNV studies [30, 33].

**Table 1:** Go enrichment results for different types of CNVR

Type of CNVRs	GO Term	Size	Count	Expected count	Enrichment value	*P*-value (FDR corrected)
DEL	Chemical synaptic transmission	278	22	8.3	2.65	0.126
DEL	Anterograde trans-synaptic signalling	278	22	8.3	2.65	0.063
DEL	Trans-synaptic signalling	279	22	8.33	2.64	0.044
DEL	Synaptic signalling	279	22	8.33	2.64	0.033
DUP	Positive regulation of adaptive immune response	32	6	0.44	13.76	0.019
DUP	Positive regulation of immune response	57	7	0.78	9.01	0.021
DUP	Positive regulation of response to stimulus	75	7	1.02	6.85	0.053
DUP	Adaptive immune response	108	9	1.47	6.11	0.018
DUP	Immune effector process	104	8	1.42	5.64	0.049
COMP	Response to stimulus	1718	45	16.63	2.71	0.000
COMP	Immune response	298	14	2.88	4.85	0.002
COMP	Detection of stimulus involved in sensory perception	477	16	4.62	3.47	0.011
COMP	B cell activation	17	4	0.16	24.31	0.013
COMP	Detection of chemical stimulus involved in sensory perception	477	16	4.62	3.47	0.014
COMP	Detection of stimulus	501	16	4.85	3.3	0.015
COMP	Immune system process	322	12	3.12	3.85	0.025
COMP	B cell receptor signalling pathway	23	4	0.22	17.97	0.027

### Population genetics of CNVRs

Population genetics analyses provide a framework to understand genetic variation seen in specific (cattle) populations. Understanding general properties of genetic variants is important, but further characterization of specific variants of interest can bring insights in recent adaptation and genome biology [35]. Although SNPs have been extensively used in characterizing various cattle populations [36], we explored the population genetic properties of CNVRs.

We focused our analyses on HOL (*n* = 315) and JER (*n* = 107) animals, derived from distinct origins and with a different breed formation history [37]. First, we coded the genotypes of our bi-allelic CNVRs (*n* = 1154 for HOL; *n* = 700 for JER) as “+/+”, “+/−”, and “−/−”. The CNVR allele frequency was classified as rare (MAF < 0.01), intermediate (0.01 ≤ MAF < 0.05) and common (0.05 ≤ MAF). In HOL, the allele frequency ranged from 0.002 to 0.29, and 5, 13, and 82% of the 1154 CNVRs were categorized as common, intermediate, and rare CNVRs, respectively. For the JER population, allele frequency ranged from 0.005 to 0.37, and 11, 20, and 69% of the 700 CNVRs were categorized as common, intermediate, and rare CNVRs, respectively.

**Fig. 3:**
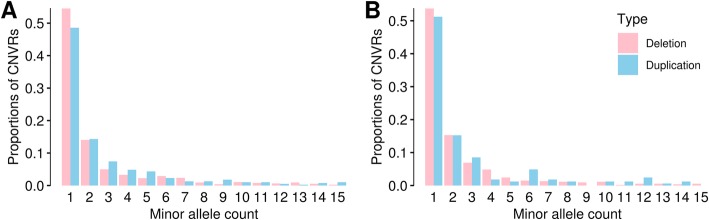
Site frequency spectrum of CNVRs. Site frequency spectra of CNVRs in HOL (**a**) and JER (**b**) population. Deletion CNVRs (pink) and duplication CNVRs (blue) are shown separately. Deletions tend to be enriched for rare CNVRs, whereas duplications tend to be enriched in common variants

**Fig. 4:**
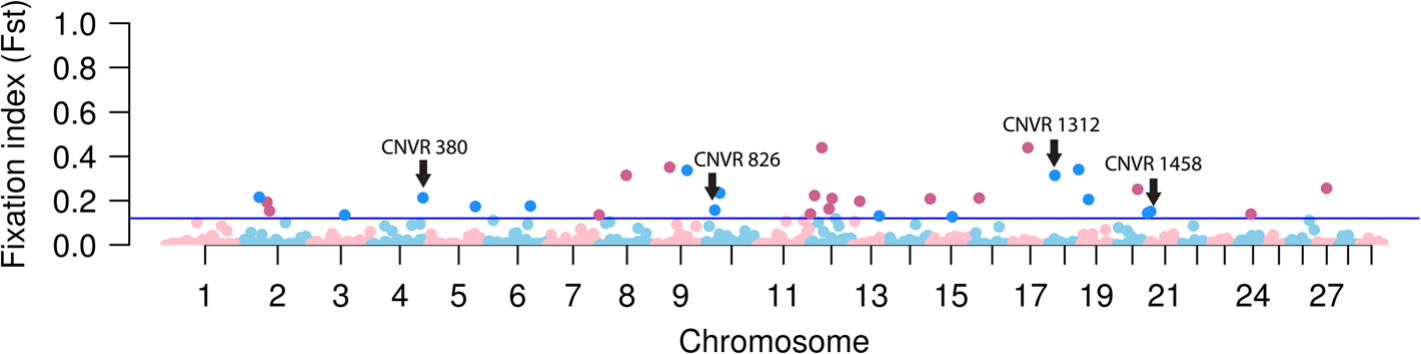
Manhattan plot for population fixation index (Fst) of CNVRs between HOL and JER**.** Population fixation index (Fst) of bi-allelic CNVRs between HOL and JER is shown in a Manhattan plot. Seventeen intergenic CNVRs (magenta) and 15 genic CNVRs (dark blue) were above the suggestive threshold (0.12; Fst > mean + 3 S.D.). CNVRs containing candidate genes are marked with arrows

We constructed site frequency spectra of CNVRs for HOL and JER separately (Fig. 3). For both populations, we observed that deletions and duplications have slightly different spectra, where deletions were more skewed towards rare CNVs, whereas duplications were observed relatively more frequent than deletions in each MAF class. We further explored the allele frequencies by applying Wright’s fixation index (Fst) [38] to characterize population structure [39] and detect loci that underwent selection [40], as done in Yali Xue et al. [41]. Given that HOL and JER have distinctive origins and breed formation history [37], we hypothesized that Fst on their CNVRs can reveal regions that underwent recent population differentiation. The Fst distribution followed an exponential decay pattern, as expected, underlining that majority of CNVRs have values close to 0, whereas only a few outliers (~ 3%) that are potentially under positive selection reached high Fst values (Additional file 2: Figure S3). We identified 32 highly diverged CNVRs (Fst > mean + 3 S.D.) of which 15 are genic and 17 are intergenic (Fig. 4 and Additional file 2: Table S6). Among the 17 intergenic CNVRs with high population differentiation (Fst = 0.12–0.44), 7 CNVRs had regulatory elements such as lncRNA and snoRNA within ~ 300 kb from the CNVRs. Among the genic CNVRs, CNVR 380 (Fst = 0.21; duplication), which is more frequent in JER (MAF = 0.24) than in HOL (MAF = 0.04), contains three genes, *CLEC5A* [42]*, TAR2R38* [43], and *MGAM*. The known functions of these genes include abnormal eating behaviour, bitter taste perception, and the synthesis of maltase glucoamylase, a starch digestive enzyme. Furthermore, CNVR 826, 1312, and 1458 overlap with genes that are known to regulate body size: *LRRC49* [44]*, CA5A* [45], and *ADAMTS17* [46–48], respectively*.* Interestingly, these CNVRs are duplications and have a high allele frequency in JER (MAF = 0.08–0.37), and a low allele frequency in HOL (MAF = 0–0.06).

Subsequently, we calculated Vst statistic, which is a widely used statistic in CNV studies [23, 49]. This statistic is analogous to Fst, but using LRR values instead of allele frequencies [28]. The Vst statistic ranges between 0 and 1, where 1 indicates population differentiation. To strengthen our confidence in the high Fst outlier regions we compared Fst and Vst statistics. Firstly, we calculated Vst for 1464 CNVRs where Fst values are available. The Pearson correlation coefficient between Fst and Vst was low (0.22), and many selection candidate CNVRs that were found privately in Vst were either driven by rare CNVRs (less than 5 copies), or with a small number of SNPs (the numbers of average SNPs for top 20 Vst CNVRs and Fst CNVRs was 3.7 and 20.7 respectively; Additional file 2: Figure S4 A-C). To correct for this, we removed CNVRs with less than 5 CNVs are called from either HOL or JER population (*n* = 1154 CNVRs). We observed that this filtering removed outlier CNVRs that were private to Vst, that were consisting of a small number of SNPs. After this filter, the 32 high Fst CNVRs were kept and the correlation coefficient was 0.52 (*n* = 310 CNVRs; Additional file 2: Figure S4 D-F). Also, CNVR 1458 which overlaps with *ADAMTS17*, showed a high Vst of 0.17 (mean Vst mean = 0.03, Vst S.D. = 0.04). Furthermore, when the copy number filter was applied to both populations, and therefore both HOL and JER had more than five copies of CNVs at each CNVRs (*n* = 44), the correlation coefficient increased to 0.81 (Additional file 2: Figure S5).

### Linkage disequilibrium of CNVRs

**Fig. 5:**
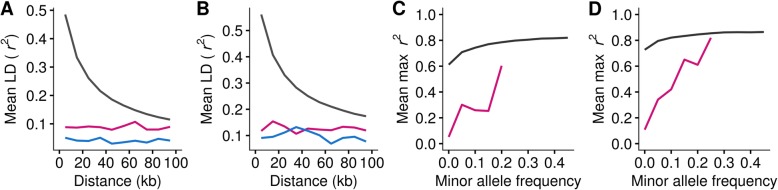
Linkage disequilibrium properties of CNVRs. Average strength of linkage disequilibrium (mean *r*^*2*^) as a function of distance from a SNP is shown for HOL (**a**) and JER (**b**). Common CNVRs (0.05 ≤ MAF) were used for the calculation; common deletion CNVRs (magenta) and common duplication CNVRs (blue) are shown together with common SNPs (black) for comparison. Taggability for HOL (**c**) and JER (**d**) was expressed as ratio of variants in high LD (*r*^*2*^ > 0.8) with SNPs within 100 kb distance. Common deletion CNVRs (magenta) and common SNPs (black) are shown in the figure. Illumina BovineHD Genotyping BeadChip SNP set was used for the LD calculation

There has been a large number of genome-wide associations (GWAS) performed using SNPs in livestock species, aiming to unravel genomic regions related to phenotypes of interest [50]. This approach exploits a large number of tagging SNPs that are in sufficient LD with causal variants. Under this framework, genetic variation caused by the causal variants is captured by the tagging SNPs, without knowing the exact causal variants. Thus, the genome-wide level of LD between SNP markers and causal variants is an important foundation of GWAS [51]. We showed that CNVRs overlap with genes more often than would be expected by chance, and that CNVs are thus likely to have an influence on phenotypes. The important follow-up question is whether the variations from CNVs are already captured by SNPs typed on commercial arrays, which are commonly used in livestock breeding programmes. We, therefore investigated pairwise LD between bi-allelic CNVRs and neighbouring SNPs on the BovineHD SNP chip. We observed generally low *r*^2^, close to zero, regardless of the distance between CNVRs and SNPs (results not shown). Subsequently, we categorized CNVRs by their allele frequency and type to investigate whether these factors influence the degree of LD. Common CNVRs have markedly higher LD (*r*^2^ = ~ 0.1 for deletion CNVRs at ~ 10 kb distance), compared to other CNVR categories (Additional file 2: Figure S6). As common CNVRs had higher LD than the rest, we compared the LD of common CNVRs with the LD of SNPs in the same MAF range (0.05 ≤ MAF < 0.29 for HOL and 0.05 ≤ MAF < 0.37 for JER). We observed distinctive difference in LD decay patterns between the CNVR-SNP pairs and SNP-SNP pairs (Fig. 5a and b). SNP-SNP LD follows a typical LD decay pattern where strong LD is observed with SNPs in vicinity and gradual decline as the distance increases, whereas CNVR-SNP LD does not follow this pattern. Also, compared to the CNVR-SNP LD (*r*^2^ = ~ 0.1 at ~ 10 kb distance), the frequency matching SNP-SNP LD was stronger (*r*^2^ = ~ 0.5 at ~ 10 kb distance). Afterwards, we used another metric, taggability, to assess LD. Taggability is the maximum *r*^2^ among the *r*^2^ values that are obtained from a variant of interest and SNP pairs. We calculated taggability for SNP-SNP pairs and CNVR-SNP pairs. For the CNVR-SNP pairs, we considered common deletion CNVRs only, as they showed the highest LD in the previous analyses. Then, mean taggability for each MAF class (bin size = 0.05) was plotted (Fig. 5c and d). The mean taggability of common deletion CNVRs is low (< 0.1) when MAF is below 0.05, and it increases as MAF increases. The SNP mean taggability follows the same pattern as shown in common deletion CNVRs. However, in spite of the similar pattern, common deletion CNVRs taggability is below the level of the SNP taggability. This shows that there is a gap in SNP taggability and CNVR taggability.

### Interesting CNVR

A large number of QTLs has been identified from various GWAS on a wide range of traits. As most GWAS have been done using SNP markers, chances are that genetic variation caused by CNVs could have been captured by QTLs that are in a high-to-perfect LD (*r*^*2*^ = ~ 1) with the CNVs. Hence, inspecting CNVRs that are in high LD with QTLs is a preliminary step to identify potentially causal CNVs. To identify candidate causal CNVs, we subset the CNVR-QTL pairs, from the total CNVR-SNP pairs, based on the QTL information from the animal QTLdb [52]. We then subset the CNVR-QTL pairs further based on *r*^*2*^, and kept high LD CNVR-QTL pairs only.

In total ~ 100,000 bovine QTLs for various traits have been reported in the animal QTL database, and we identified 2519 QTLs to be paired with 679 CNVRs within a distance of 100 kb in the HOL population. Among these, CNVR 547 (BTA6:84,395,081-84,428,819, deletion, MAF = 0.24) had the highest LD with 13 QTLs (average *r*^*2*^ = 0.59; max *r*^*2*^ = 0.74). The 13 QTLs were associated with casein proteins, which constitute four out of six bovine milk proteins. The four genes coding for the casein proteins are located in the so called casein cluster, which is ~ 1 Mb distant region from CNVR 547 (BTA6:85.4–85.6 Mb). Given the degree of LD for CNVR 547 and the QTLs that is lower than perfect linkage, it is unlikely that the CNVR 547 is the causal variant for the casein protein traits. Nevertheless, CNVR 547 was an interesting variant as it was private to in HOL population with high MAF (0.24), and was close to the casein cluster that are highly relevant for dairy production.

Assuming that CNVR 547 is not the causal variant for the casein traits, a possible explanation for the high MAF can be selective sweeps. Selective sweeps increase allele frequencies of neutral variants that are in LD with the selection target variant, which in this case probably is the casein cluster. Two studies of Holstein populations support this hypothesis. Firstly, one selective sweep study in a German Holstein population revealed an extended range of LD in haplotypes that contain the casein cluster [53]. Secondly, GWA study on casein traits in a Danish Holstein population identified a broad GWAS peak (BTA6:60–100 Mb) that contains the casein cluster [54]. The broad GWAS peak also indicate high LD in this regions, that matched with the findings from Qanbari et al. [53]

Another explanation for the high MAF of CNVR 547 might be the direct selection on the variant itself. For instance, CNVR 547 overlaps with the *UGT2B4* gene, which is involved in the detoxification pathway of exogenous compounds [55]. To see whether CNVR 547 overlaps with regulatory elements, besides overlapping with the upstream region of the *UGT2B4* gene directly, we called promoters and enhancers from ChipSeq data from Villar et al. [56]. CNVR 547 overlaps not only with the upstream (a start codon and the first two exons), but also with the enhancer of *UGT2B4* (BTA6: 84,413,246-84,413,740), and is thus likely to disrupt the function of the *UGT2B4* gene. To summarize, our analyses imply that a high MAF of CNVR 547 might be due the selective sweep in the casein cluster or the consequence of direct selection on CNVR 547 itself due to the functional impact of the overlap with *UGT2B4* and its enhancer. Nonetheless, we cannot exclude drift as a possible driver for the high allele frequency of the CNVR 547.

## Discussion

In this study, we discovered CNVs using bovine high density SNP array data. Using CNVRs that are constructed using the CNVs, we reported the functional impact and population genetic features of the CNVRs. They are further discussed below.

### CNV discovery in the genome build ARS-UCD1.2

We observed different CNV discovery results between UMD3.1 and ARD-UCD1.2. The different results were to be expected, given the different sequencing platforms used for the assemblies. Long-read sequencing platforms are shown to perform better in retrieving repeat regions, which is considered to be challenging in short-read sequencing [57]. Among others, the most intriguing difference was observed for the BTA12:70–77 MB region. Based on the changes in BovineHD SNPs between UMD3.1 and ARS-UCD1.2, we postulated that the two genomes assemblies differ in this regions largely. Subsequently, the changes in the genome assemblies led to different CNV discovery results. We, then, further postulated that this region (BTA12:70-77 Mb in UMD 3.1) might contain repeated sequences, rather than the reported CNV, for two reasons. Firstly, the SNP density in this region is a quarter of the genome-wide average SNP density in UMD3.1 (71 SNPs/Mb and 292 SNPs/Mb, respectively; Additional file 2: Figure S2). SNP probes in repeat regions can reduce specificity of hybridization, and hence are often filtered out during SNP probe selection [58–60], which can explain why some regions show a sharp decrease in SNP density. Secondly, SNP probes in segmental duplications (sequence identity > 90%) can induce confounded deletion calls due to cross-hybridization of paralogous sequences [61]. Our data set based on UMD3.1 was indeed enriched for a large number of deletion calls in this region. We regard this large difference as evidence underlining the importance of the quality of the reference genomes and the impact this has on CNV calling results.

### Functional impact of CNVRs

In our functional impact analyses, we showed that the overlap between genes and CNVRs is higher than the overlap in a neutral scenario. This finding is in line with human and rat CNV studies, which showed that the overlap between CNVs and genes is significantly higher than expected by chance [62–64]. These studies were based on medium-to-large size human CNVs, and rat CNVs were found from exome arrays (CNV length ranged between 5 and 256 kb). However, more recent studies, based on a finer resolution of CNVs, concluded that CNVs are biased away from genes and functional elements [5, 65–67].

Also, we observed that MAF have impact on different types of overlap between genes and CNVRs. In our findings, common deletion CNVRs were biased away from the genic regions, yet the common duplication CNVRs were skewed toward the genic part. This was contradicting with findings from another study, which showed both common deletions and duplications are skewed away from genic part [65].

We assume that these conflicting findings might arise from a curation of SNP array based CNVs in our dataset, which is affected by an ascertainment bias. An ascertainment bias of SNPs in commercial arrays can introduce a two-fold bias in CNV discovery. Firstly, the SNP density of a given array will constrain the size of CNVs that can be discovered. Secondly, SNP probes are designed such that complex regions, such as segmental duplications (SD), are under-represented [61, 68]. The SNP density of BovineHD BeadChip array in unique regions is 292 probes/Mb, whereas it drops to 95 probes/Mb in SD regions, showing a 67.5% reduction. Based on this, we speculate that the uneven genome-wide SNP coverage might introduce a systematic bias in CNV discovery. Taken together, the studies that focused on mid-sized CNVs [62–64] are in line with our findings, whereas studies based on tiling oligonucleotide microarrays [65] and whole genome sequencing data [5, 66], which can provide rather complete genome-wide coverage with a various size range of CNVs [4], show different results.

Furthermore, another layer of bias in CNV discovery using SNP array is that discovery of duplication is less sensitive than that of deletions. Consequently, most small CNVs are overwhelmingly deletions, whereas duplications usually are discovered based on relatively large number of SNPs than deletion, which makes duplications longer than deletions [4]. Indeed, in our CNV discovery, we found two folds more deletions than duplications (9171 vs. 4101), and the mean length of duplication was longer (44.2 kb vs 74.6 kb). This deletion-duplication bias might explain why common duplication CNVRs in our dataset are more likely to affect genic region compared to the rare duplication CNVRs, whereas it was the opposite in a study mentioned above [65].

The need to re-evaluate the functional impact of CNVs, as CNV detection resolution became finer, along with the advancement in assay technologies and detection algorithms, was already pointed out [68]. Moreover, a recent study exploiting long-read sequencing data detected 237 and 34% more insertions and deletions, respectively, compared to known variants detected from short-read sequencing data [69]. Taken together, the CNVs discovered in our dataset (> 1 kb) were shown to be biased towards genic regions. However, we stress the need of re-visiting CNVs with finer resolution and unbiased genome-wide coverage, to fully comprehend their functional consequences in cattle genomes.

### Population genetics of CNVRs

We explored the population genetics of CNVRs by examining the site frequency spectra and Fst. The frequency spectra differed for deletion CNVRs and duplication CNVRs. Given the skewed number of rare deletions and common duplications, we corroborate that deletions might be under stronger purifying selection. Nevertheless, as explained earlier, inherent bias in CNVs from SNP array (deletion discovery is more sensitive than duplication discovery), we cannot entirely exclude a possibility that the differed frequency spectra might be an artefact.

Furthermore, we used Fst to identify CNVRs that are highly diverged. Among the 32 CNVRs that pass the threshold, of which 7 intergenic CNVRs had regulatory elements in neighbouring regions. This finding underlines that potential recent positive selection probably acted on regulatory elements. Among the 17 genic CNVRs, we identified CNVRs that overlap with interesting candidate genes. The CNVR 380 overlaps with *CLEC5A*, *TAR2R38* and *MGAM* gene that are related to taste perception and a digestion enzyme, maltase. One selective sweep study revealed that a region containing *TAR2R38* and *MGAM* is highly diverged between dogs and wolves. Dogs produce a longer form of maltase than wolves, due to a 2 bp deletion that disrupts the stop codon, and the same mutation was also seen in herbivore species (rabbits and cows) [70]. The longer form of maltase might be the consequence of adaptive evolution in response to a starch-rich diet during dog domestication. Given that the partial duplication of *MGAM* can lead to increased length of maltase, a high duplication frequency seen in the JER population (MAF = 0.24) might be a hint that feed related adaptive evolution occurred in the JER population. Also, we identified genes related to body size (*LRRC49* in CNVR 826, *CA5A* in CNVR 1312, and *ADAMTS17* in CNVR 1458). Among these genes, *ADAMTS17* has been reported as one of the height determining genes in various species, such as cattle, horse, and human [46–48]. Also, a deletion variant overlapping with *ADAMTS17* was shown to be highly diverged between HOL and JER in a previous study [66]. Given that CNVR 1452 we found is a duplication locus, it might be a different mutation than the one found by Mesbah-Uddin et al. [66]. Nonetheless, our and the previous findings revealed that CNVs overlapping with *ADAMTS17* gene to be diverged between HOL and JER. This supports *ADAMTS17* gene as a candidate gene that can explain the phenotypic differences (i.e. body size) between the two breeds.

Additionally, we used Vst analyses to confirm the selection candidate CNVRs based on Fst analyses. The preliminary results from Vst statistic from 1464 bialleleic CNVRs showed that extreme Vst could be obtained from very rare CNVs (less than 5 CNVs observed) and short-sized variants. We consider correcting for these factors in analysing Vst statistic is crucial, as it could reduce falsely derived selection signal from false positive singletons [24]. We have seen that overall concordance between Vst and Fst was 0.52, when rare CNVRs (number of CNVs < 5) were filtered out in either of the populations. Furthermore, when rare CNVs were filtered for both of the populations, which means CNVRs were present in both populations with more than 5 copies, the correlation coefficient was 0.81. This number is slightly lower than 0.9, which was shown in human CNV study [49]. These findings underline high concordance of Fst and Vst when CNVRs are present in both populations with sufficient MAF. Thus, although we could obtain Vst confirmation for CNVR 1458, which overlaps with *ADAMTA17*, we could not obtain such confirmation for CNVRs that are at low MAF in either of the two populations.

### Linkage disequilibrium of CNVRs

To summarize our findings on LD properties of CNVRs, CNVRs are generally in low LD with SNPs, and CNVR taggability is lower than SNP taggability, which indicates a taggability gap. However, findings on the taggability are conflicting. Although some studies reported high CNV taggability [65, 68, 71–73], as high as SNP-SNP taggability, some studies reported low CNV taggability [28, 61, 68, 74, 75] as shown in our results. The taggability gap can be explained by three factors. Firstly, LD is affected by allele frequency. High LD can be obtained when the allele frequencies of the two loci match [76]. Van Binsbergen et al. (2014) empirically showed that SNP-SNP pairs with small MAF difference (< 0.05) had high predicted LD (*r*^*2*^ > 0.8) using WGS data [77]. In our dataset, the majority of CNVRs is at low allele frequency (88 and 95% of CNVRs in JER and HOL are at MAF ≤ 0.05), whereas BovineHD SNPs are biased away from rare MAF (10% of SNPs are at MAF ≤ 0.05). Thus, the allele frequencies of CNVRs and SNPs were largely unmatched, which can be explain the low LD. Secondly, deletions are tagged better than duplications. Even studies that found high taggability for common deletions, only found relatively poor taggability for duplications [5, 65, 71]. This might be due to dispersal duplications [78], which relocate the duplicated segment of DNA in a different haplotype background than the “parental locus” [79]. Thus, the LD of duplications might be lower than that of deletions. Lastly, local SNP density can influence the level of LD. Redon et al. [28] and Locke et al. [75] suggested that a paucity of SNPs in repeat-rich regions to serve as potential tags can be an explanation for the taggability gap. Indeed, Cooper et al. [61] and McCarroll et al. [68] used different SNP sets in their CNV LD analyses. The first SNP set was HapMap phase 2 SNP set, which is known to cover the whole genome uniformly (~ 3.1 M probes). Next to this, they used SNP sets obtained from commercial SNP arrays, which have uneven SNP density along the genome (550 K ~ 1 M probes). They found that ~ 80% of CNVs are in high LD (*r*^2^ > 0.8) when HapMap phase 2 SNP set was used, whereas ~ 50% of CNVs were in high LD with the commercial array SNP sets.

Based on our and previous findings, we postulate that LD between common deletion CNVRs and SNPs is not necessarily low. However, we could not obtain high LD with our CNVRs, because our CNVRs were skewed towards rare MAF. The MAF difference between CNVRs and SNPs can explain lower LD shown in rare CNVRs, compared to common CNVRs. However, as shown in another study [24], singletons found from PennCNV software could be false positives, which could lead to low LD as well. Thus, we could not exclude the possibility that the low LD in rare CNVRs was partly caused by false positive singleton CNVs driving low LD. Also, BovineHD SNPs were underrepresented in SD regions, where SNP probe design is difficult due to high sequence identity. Deprivation of SNPs in these regions probably led to lack of markers that can serve as tagging markers. Follow-up research using a SNP set that uniformly covers the whole bovine genome might unravel more complete LD properties of CNVs.

### Interesting CNVR

Furthermore, in search of CNVRs that are causal variants of traits, we investigated CNVRs that are in high LD with known QTLs. CNVR 547 was shown to be in high LD with casein QTLs, although it was below perfect linkage, thus unlikely to be the causal variant. However, this opened up an interesting avenue to see the CNVR 547 in light of LD and selection. We proposed three possible explanation for CNVR 547 to reach high MAF:1) selective sweeps, 2) direct selection on CNVR 547 that affects the enhancer of *UGT2B4* gene, and 3) drift. Although we could not unravel how CNVR 547 has reached high MAF in the current study, we deem it as an interesting case, which a CNVR can be understood in population genetics theories, such as selective sweeps and drift. Also, we had a limited number of CNVRs obtaining high LD with QTLs. This was partially due to because most CNVRs were rare, thus predisposed to have low LD. Therefore, re-visiting CNVR-QTL pairs, based on CNVs that are detected from a different platform (i.e. WGS) might reveal more candidate CNVs that might be the underlying causal variants of traits.

## Conclusions

In this study, we discovered CNVs in bovine genomes and explored their functional impact and population genetics features. Using commercial high-density SNP arrays, we identified 14,272 CNVs, that built 1755 CNVRs (cover ~ 2.8% of the bovine autosomes), and the CNVRs were further used as genetic loci this study. In the functional impact analyses, we showed that CNVRs are likely to have functional impact based on their overlap with genes. Also, we investigated CNVRs in light of population genetics. We identified 32 highly differentiated CNVRs between HOL and JER based on Fst values. Two of the highly diverged CNVRs overlapped with the *ADAMTS17* gene and *MGAM* gene, which are involved in body size and starch digestion enzyme, respectively. In the LD analyses, CNVR-SNP LD was lower than SNP-SNP LD, mainly due to low MAF in CNVRs and uneven SNP density.

These findings together impose several implications for future CNV studies. The first implication is about the functional impact of CNVs. SNP based GWAS is a commonly used design to find functional SNPs that are associated with traits. Given the low CNVR-SNP LD, SNP based GWAS are unlikely to detect CNVRs with functional impact. Consequently, GWA studies that associate CNVRs and traits directly can add valuable insights into understanding economically important traits. Secondly, the low CNVR-SNP LD implies that the majority of CNVRs in our study is probably not captured in the current genomic prediction, where SNP markers are used. Thus, we underline the importance of follow-up studies on investigating methods to include CNVs in genomic prediction and evaluating the usefulness of CNVs in improving the accuracy of genomic prediction.

## Methods

### Animal samples and ethics

The study population consisted of two dairy cattle breeds, 331 Holstein Friesian (HOL), 115 Jersey (JER), as well as 29 crossbreds of HOL and JER. Among these, 18 HOL and 17 JER animals were cows and the rest were bulls. All samples were genotyped using an Illumina BovineHD Genotyping BeadChip (Illumina, San Diego, CA, USA), which contains 777,692 SNPs. All of these genotypes are owned by commercial dairy breeding company CRV (Arnhem, the Netherlands). The Genotype data was provided by CRV.

### Identification of CNVs

We identified CNVs using PennCNV software [27] which exploits a Hidden Markov Model algorithm. For each individual, log R ratio (LRR) and B allele frequency (BAF) per SNP were inferred using the Illumina Genome Studio software package (Illumina, San Diego, CA, USA). Autosomal SNPs of BovineHD Genotyping BeadChip (*n* = 735,965; Illumina, San Diego, CA, USA) were used, and their positions were based on the genome assembly ARS-UCD1.2. We called CNVs in 29 Bovine autosomes. The waviness in LRR values caused by GC contents were adjusted afterwards. We chose PennCNV software, together with BovineHD Genotyping BeadChip, as this method showed high confirmation based on qPCR validation in a previous study (91.7% for CNVs found in multiple animals and 40% for singleton CNVs) [24]. After the initial CNV detection, poor quality individuals (*n* = 13) were filtered out with the default criteria suggested by the developer of the PennCNV software (LRR standard deviation > 0.30, BAF standard deviation > 0.001 and Waviness factor > 0.05). Afterwards, the distribution of the number of CNVs per individual was inspected using QQ plots (Additional file 2: Figure S7). The distribution was continuous until 100, and individuals with more than 100 CNVs largely deviated from the distribution (*n* = 10). The same filter on the distribution of the total length of CNVs per individual was applied and identified outlier samples (*n* = 11). These two filter steps identified 11 outlier individuals (among the 11 outlier animals identified in the second filter, 10 were identified as outliers in the first filter), and subsequently these individuals were removed to prevent the introduction of a large number of possible false positive CNVs. Lastly, we merged two adjacent CNVs that have the same copy number state, when the gap between the two CNVs was less than 10% of the total length, using the clean_cnv.pl script provided by PennCNV software, which resulted in 451 individuals with 14,272 CNVs in the combined dataset of the two breeds.

### Constructing CNVRs

The CNVs were aggregated into CNV regions (CNVR) based on 1 bp overlap, following Redon et al. (2006) [28]. CNV regions that exclusively contain deletions or duplications were classified as deletion CNVRs and duplication CNVRs and treated as bi-allelic loci. In case of CNVRs that consisted of both deletions and duplications, we defined them as complex CNVRs. The CNVRs were compared together with SD and SNP density in. The SDs detected by Feng et al. [80] based on UMD3.1 were remapped to ARS-UCD1.2 using NCBI Genome Remapping Service. Afterwards, the density of SDs and SNPs were calculate for 5 Mb bin using BEDtools [81]. Circos software [82] was used to visualize CNVRs, SD density, and SNP density. The length of CNVRs and SD was log transformed for the circular plot.

### Assessment of CNV discovery results

We repeated the same CNV calling steps using 735,293 autosomal SNPs based on the genome assembly UMD3.1. After the initial CNV detection, the same quality control filters were applied as explained above. The default criteria filtered out 18 individuals, and another 11 outliers detected from QQ plots of the number of CNV per individual and the total length of CNV per individual were removed (Additional file 2: Figure S7). Subsequently, split CNVs that have small gaps in between were merged as described for ARS-UCD1.2. From the 447 individuals that passed the quality control criteria, 24,264 CNVs were called, and 1866 CNVRs were constructed as explained above. Finally, we compared the CNVs and CNVRs between the two different genome assemblies, UMD3.1 and ARS-UCD1.2, in terms of number and length.

### Functional impact of CNVRs

The CNVRs were overlapped with gene annotations using Ensembl Variant Effect Predictor [83] (Cow release 95) to explore their functional impact. Subsequently, CNVRs were classified depending on their functional impact, as done in Conrad et al. [65]. First, we identified intergenic CNVRs, which did not overlap with genes, and genic CNVRs which overlapped with genes. Among the genic CNVRs, ones containing a complete gene or genes were classified as “whole gene”. Genic CNVRs that overlapped with some part of genes were further classified as “intronic”, when CNVRs overlapped with introns exclusively; as “stop codon”, when CNVRs overlapped with stop codon; as “promoter region”, when CNVRs included promoter region (500 bp from transcription start site). The remaining CNVRs that overlapped with an exon or exon(s) and intron(s) were considered as “exonic”. In the case of CNVRs overlapping with more than one gene, and thus having more than one category assigned, (i.e. that contains a complete gene and also a promoter region of another gene), we assigned one unique category in the following order: 1) whole gene, 2) stop codon, 3) promoter region. With the steps explained above, each CNVR was assigned a unique category. Then, we investigated whether the functional impact classes were influenced by type of CNVRs (1125 deletion, 513 duplication, 117 complex CNVRs). Also, the influence of allele frequency on the functional impact classes were analysed and the allele frequency classes were defined as common (MAF ≥ 0.05 in any population, 56 CNVRs), intermediate (0.1 > MAF ≥ 0.01, 267 CNVRs), and rare (MAF < 0.01 in HOL and JER, 115 CNVRs). To see whether the functional impact category differs significantly depending on type of CNVRs and MAF classes, Pearson’s Chi-square tests were performed. Afterwards, CNVRs were classified depending and type and allele frequency in HOL and JER separately and the overlap with functional classes were analysed. Afterwards, we performed permutation tests to understand whether the observed overlap between CNVRs and a genomic feature is high or low, compared to random genomic regions. The permutation tests were performed with the R package “regioneR” [84]. We generated a random set of regions in the genome, with the same number and length of genomic features, and did this 1000 times. For each permutation, the number of overlaps between random CNVRs and the genomic features was recorded and then used to estimate the expected number of observations. The observed and the expected numbers of overlaps were then tested for significance (z-test). Subsequently, the PANTHER classification system [85] was used to perform gene ontology enrichment tests for the genes that overlapped with CNVRs. All known bovine genes (Ensembl release 95) were used as a reference set to test whether the CNVR overlapping genes were enriched for or deprived of a specific biological process, cellular composition, and molecular function, with False Discovery Rate correction (α < 0.05) for multiple tests.

### Population genetics of CNVRs

We explored bi-allelic CNVRs in HOL and JER in light of population genetics. We genotyped bi-allelic CNVRs in HOL and JER into “+/+”, “+/−”, and “−/−”, following McCarroll et al. [72]. These genotypes were used to calculate the allele frequency of each CNVR locus. Subsequently, we constructed site frequency spectra in HOL and JER to understand selection pressure on CNVRs. Wright’s population differentiation index (Fst) [38] was used to investigate recent divergent selection in HOL and JER populations. Fst was calculated for 1471 bi-allelic CNVRs, using PLINK (version 1.9., http://pngu.mgh.harvard.edu/purcell/plink/) [86].

### Linkage disequilibrium of CNV

We estimated the degree of LD between bi-allelic CNVRs and SNPs by calculating *r*^2^ in the JER and HOL populations, respectively. To have a reference, we also estimated SNP-SNP LD, limited to SNPs with the same MAF range as common CNVRs (0.05 < MAF < 0.30 for JER and 0.05 < MAF < 0.24 for HOL). The SNPs inside the CNVRs were masked to prevent a bias introduced during the phasing step, as done in Conrad et al. [65]. SNPs with low minor allele frequency (MAF < 0.001), with low call rates (< 90%), or with deviations from the Hardy–Weinberg equilibrium (*P* < 1e^− 9^) were removed. For CNVRs, the same filters were applied, except the call rate criteria. Phasing was done with Shapeit [87] and the *r*^2^ values of CNVR-SNP pairs within a 100 kb distance were calculated in PLINK (version 1.9., http://pngu.mgh.harvard.edu/purcell/plink/) [86]. Afterward, QTLs that were shown to be significant in association studies were downloaded from Animal QTLdb [52] (release 37) and intersected with the CNVR-SNP pairs to see whether CNVRs are in high LD with known QTLs. To overlap the CNVR 547 and functional elements in bovine genomes, we used the data from Villar et al. [56]. We downloaded the ChipSeq data and aligned them to ARS-UCD1.2 using BWA-MEM (0.7.15) [88], and called the enhancers and promoters as explained in the original paper.

## Supplementary information

**Additional file 1: Figure S1.** Distribution of CNV length. **Figure S2.** Circular map of autosomal CNVRs in UMD3.1. **Figure S3.** Distribution of Fst values. **Figure S4.** Vst-Fst plots for 1464 biallelic CNVRs and after filtering for minimum of five copies of CNVs per CNVR in either of HOL and JER population. **Figure S5.** Vst-Fst plots after filtering for minimum of five copies of CNVs per CNVR in both HOL and JER populations. **Figure S6.** Linkage disequilibrium of CNVRs in different MAF classes. **Figure S7.** QQ plots for CNV quality control.

**Additional file 2: Tables S1.** Summary statistics for CNVs in ARS-UCD1.2 and UMD3.1. **Table S2.** Detailed features of CNVRs on autosomes identified in this study. **Table S3.** Chromosome-wide CNVR coverage based on ARS-UCD1.2. **Table S4.** Chromosome-wide CNVR coverage based on UMD3.1. **Table S5.** Comparison between CNVRs identified in the present study with previous studies in terms of count and length. **Table S6.** CNVRs with high Fst values (0.12 <) and the genes affected by the high Fst CNVRs.

## Data Availability

The data that support the findings of this study are available from CRV B.V. (Arnhem, the Netherlands) but restrictions apply to the availability of these data, which were used under license for the current study, and so are not publicly available. Data are however available from the authors upon reasonable request and with permission of CRV B.V.

## Abbreviations

CNV: Copy number variation
CNVR: CNV region
Fst: Fixation index
HOL: Friesian Holstein
JER: Jersey
LD: Linkage disequilibrium
MAF: Minor allele frequency
QTL: Quantitative trait loci
SD: Segmental duplications
SNP: Single nucleotide polymorphism
