# Supplementary material for: Functional and population genetic features of copy number variations in two dairy cattle populations
Source: BMC Genomics. 2020 Jan 28;21:89. doi: 10.1186/s12864-020-6496-1 (PMC6988284; doi:10.1186/s12864-020-6496-1)
Supplement: Supplementary file 1 — Additional file 1: Figure S1. Distribution of CNV length. Figure S2. Circular map of autosomal CNVRs in UMD3.1. Figure S3. Distribution of Fst values. Figure S4. Vst-Fst plots for 1464 biallelic CNVRs and after filtering for minimum of five copies of CNVs per CNVR in either of HOL and JER population. Figure S5. Vst-Fst plots after filtering for minimum of five copies of CNVs per CNVR in both HOL and JER populations. Figure S6. Linkage disequilibrium of CNVRs in different MAF classes. Figure S7. QQ plots for CNV quality control. [file 12864_2020_6496_MOESM1_ESM.docx]

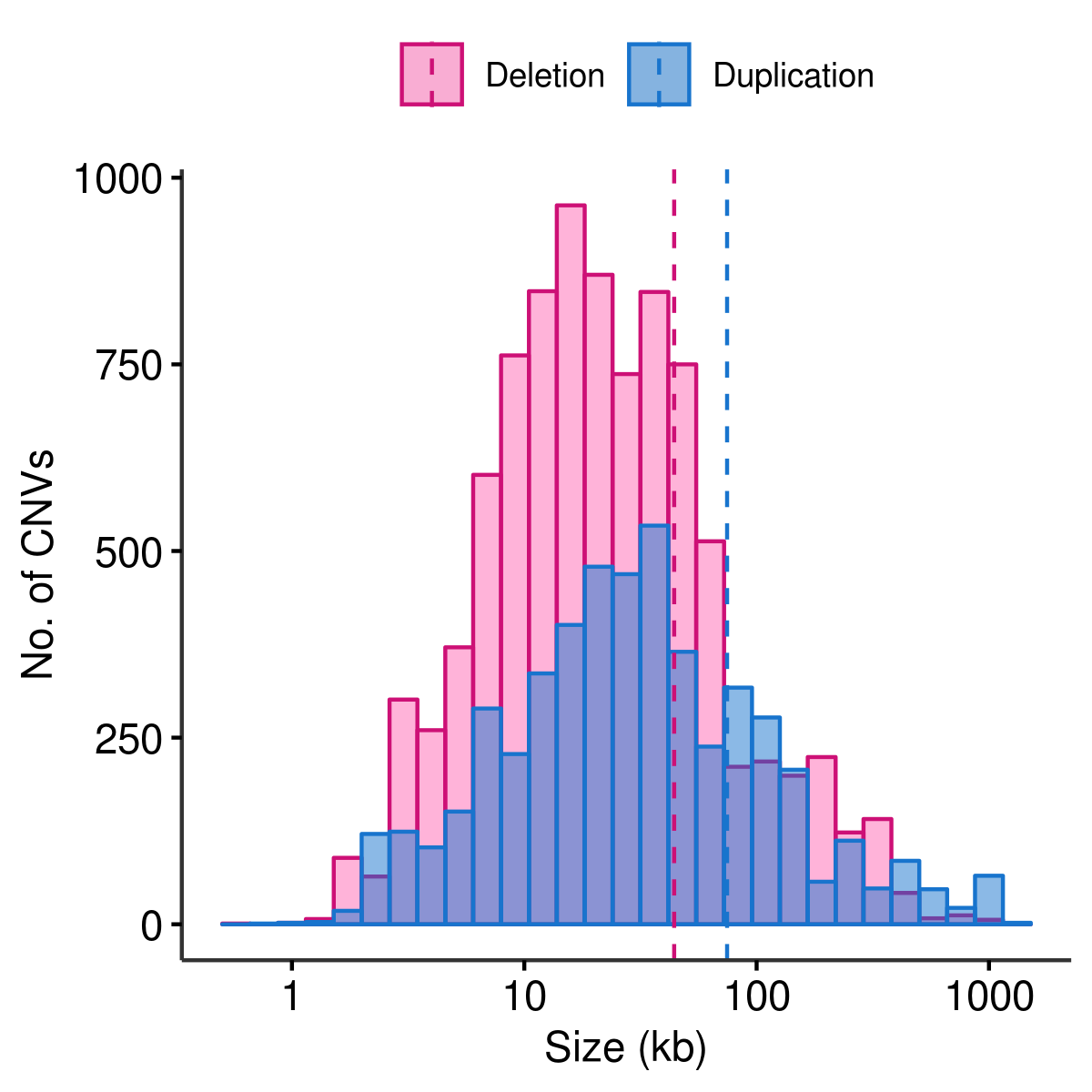
**Figure S1. Distribution of CNV length**

The CNV length distribution for deletions (pink) and duplications (blue). The dashed line showed the mean length for deletions (pink; 44.1 Kb) and duplications (blue; 74.6 Kb)

**Figure S2. Circular map of autosomal CNVRs in UMD3.1**


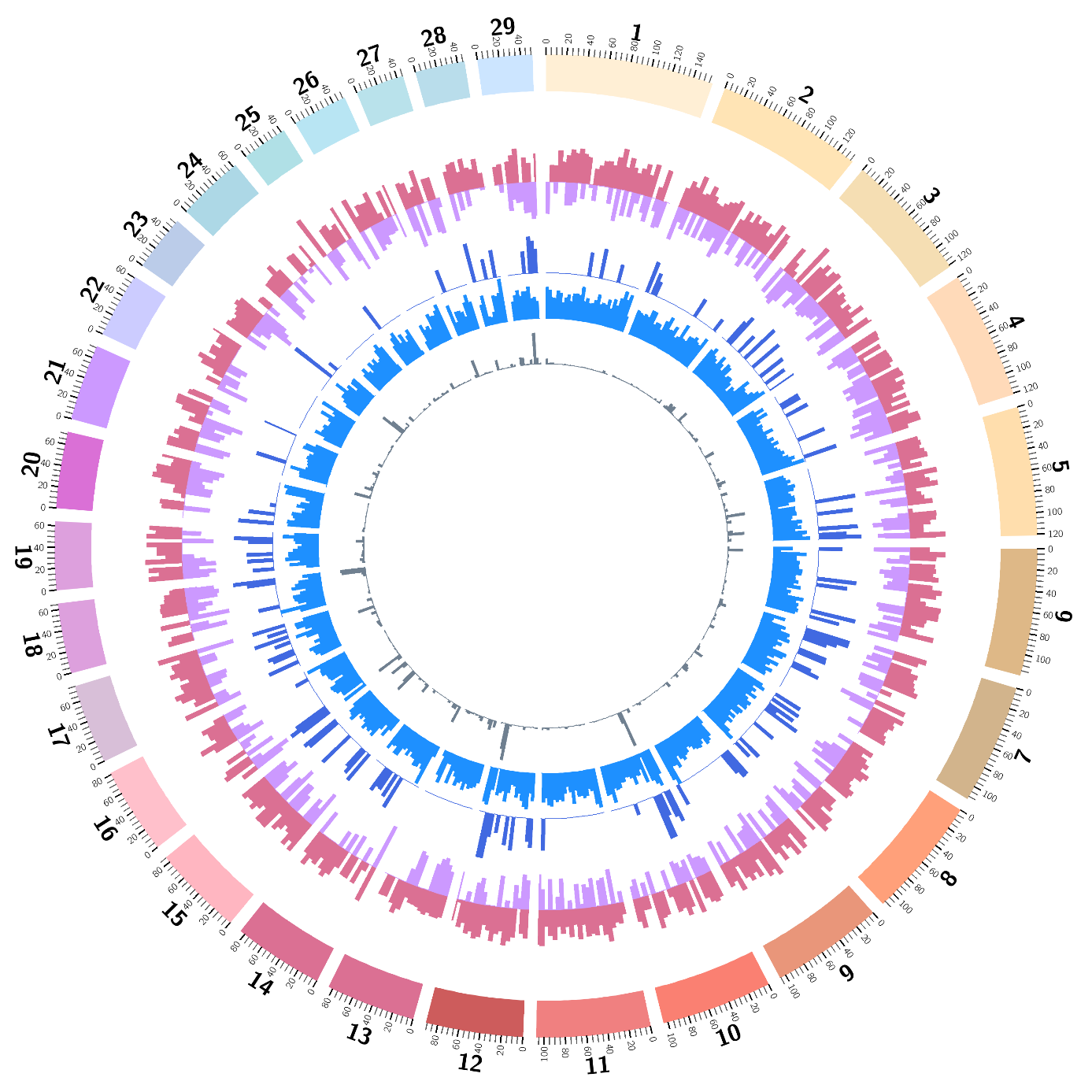


From the outside to the inside of the external circle: chromosome name; genomic location (in Mb); histogram representing density of deletion CNVRs in 5 Mb bin (pink); histogram representing density of duplication CNVRs in 5 Mb bin (purple); histogram representing density of complex CNVRs in 5 Mb bin (blue); number of BovineHD BeadChip array SNPs in 5 Mb bin (dark grey); histogram representing density of segmental duplications in 5 Mb bin (light grey).

**Figure S3. Distribution of Fst values**


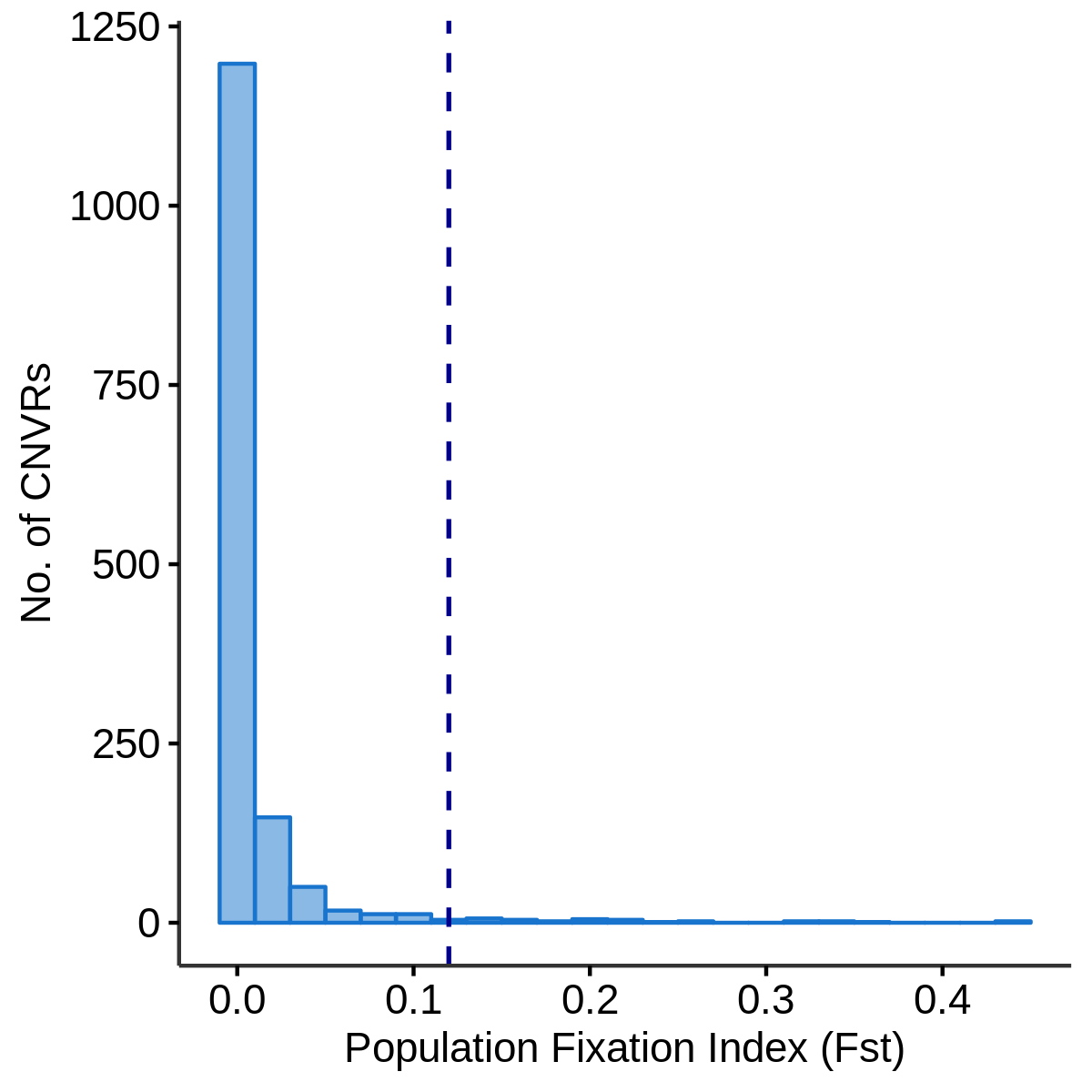


The distribution of population Fixation index (Fst) of 1,464 bialleleic CNVRs. Majority of them have values close 0, and a small number of CNVRs have Fst value larger than 0.12, which is shown in the dashed line (the threshold was mean + 3 S.D.)

**Figure S4. Vst-Fst plots for 1,464 biallelic CNVRs and after filtering for minimum of five copies of CNVs per CNVR in either of HOL and JER population**


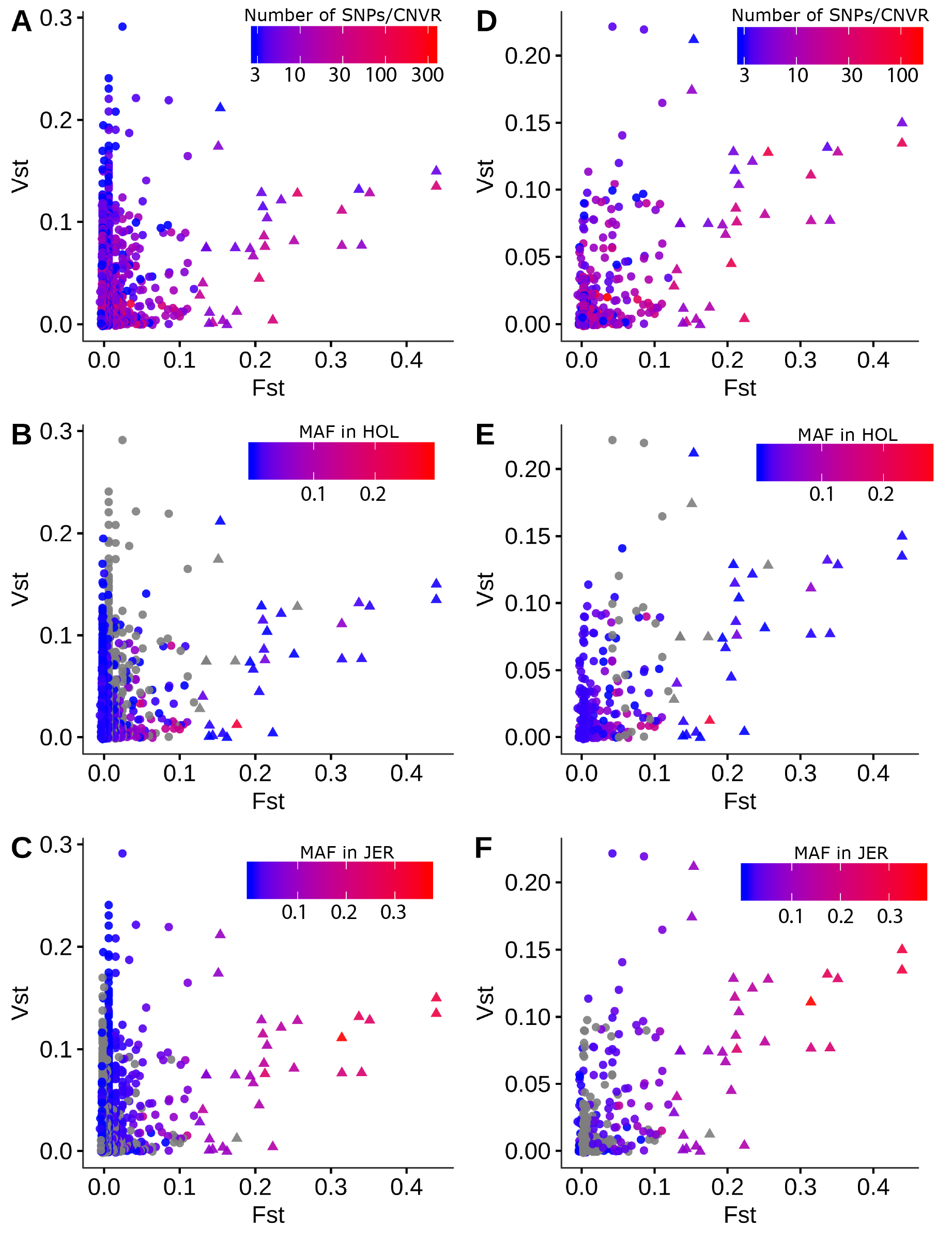


Panel A-C show Vst-Fst plots for 1,464 biallelic CNVRs, with the colour scale with SNP numbers per CNVR, MAF in HOL, and MAF in JER, respectively. Panel D-F show Vst-Fst plots for 310 CNVRs that passed the filter for minimum of 5 copies in either of the two populations. the colour scale is same as the panel A-C. Dots with triangle shapes are CNVRs passed high Fst threshold (0.12). Dots shown in grey are CNVRs that are not variant in a given population (i.e. grey dots in panel B indicate CNVRs that are not variant in HOL). The correlation coefficient of Vst-Fst was 0.22 for panel A-C, and 0.52 for panel D-F.

**Figure S5. Vst-Fst plots after filtering for minimum of five copies of CNVs per CNVR in both HOL and JER populations**


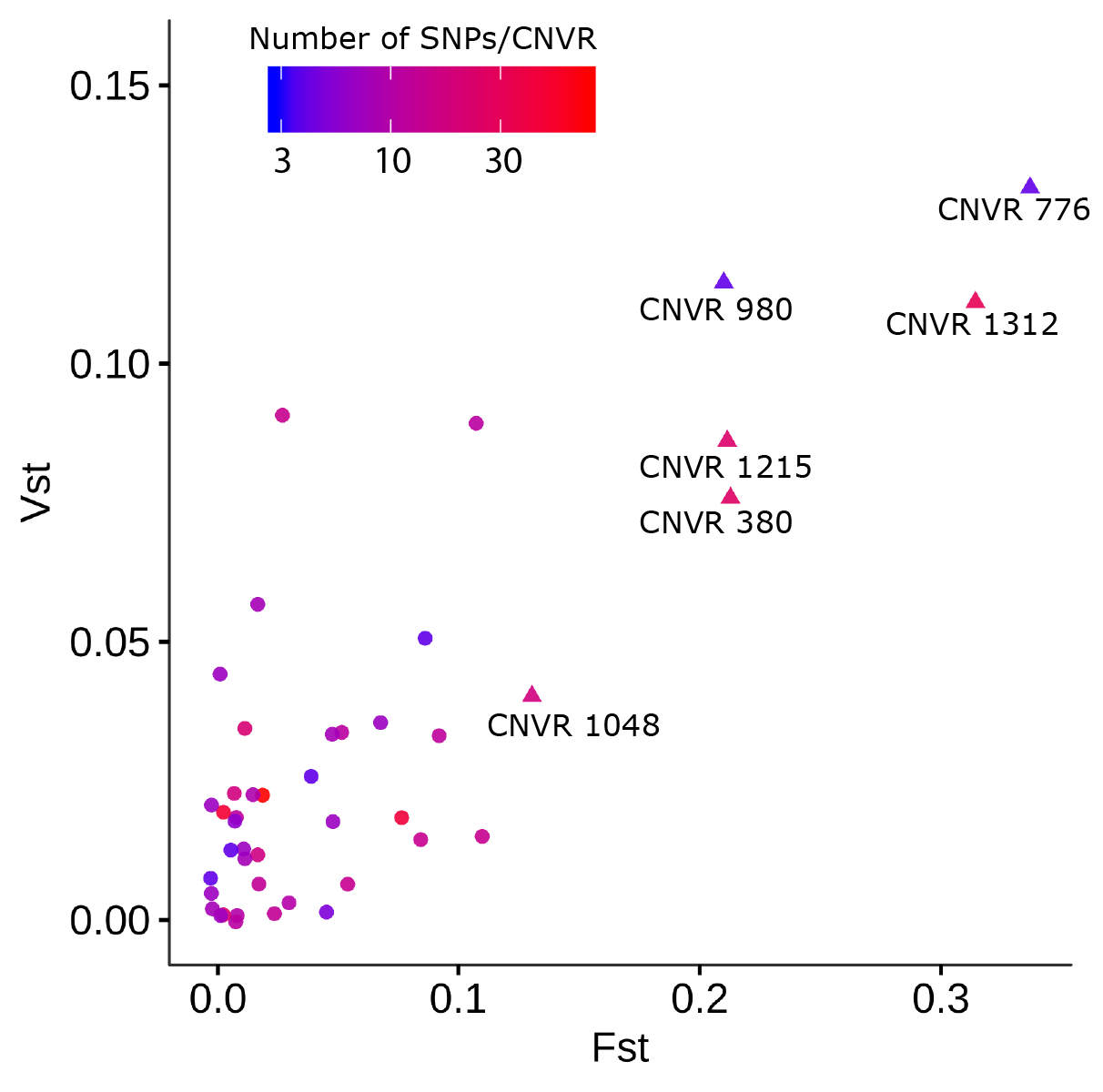


Vst-Fst plot for 44 CNVRs that have at least five CNVs in both HOL and JER populations. This filtering step removed 26 high Fst CNVRs from the 32 high Fst identified. The correlation coefficient was 0.81.

**Figure S6. Linkage disequilibrium of CNVRs in different MAF classes**


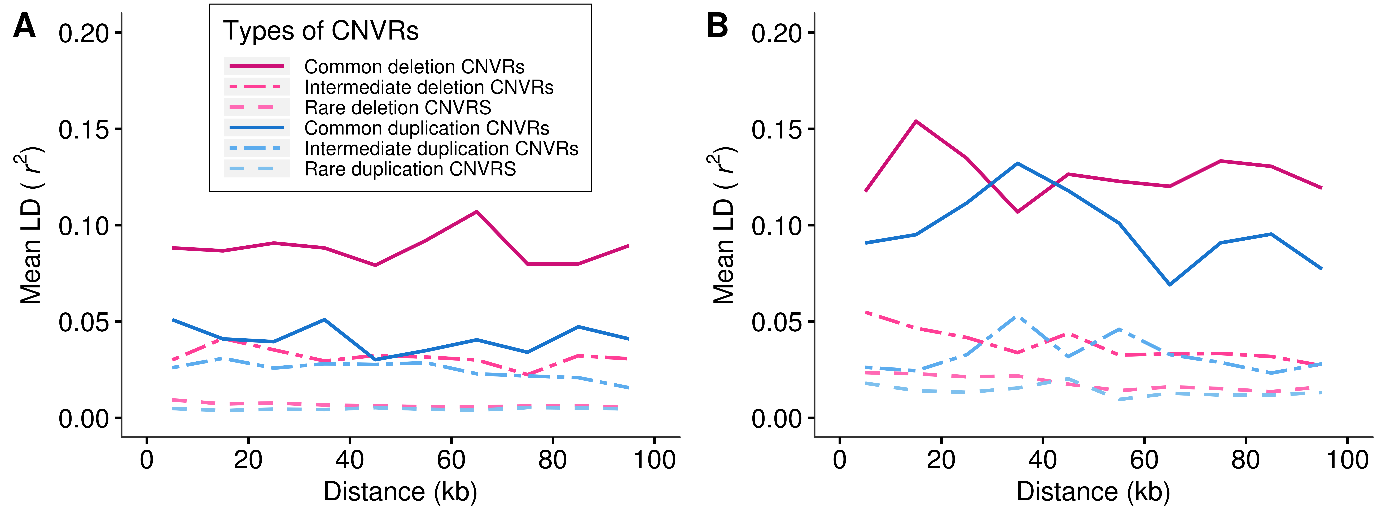


Average strength of linkage disequilibrium (mean *r^2^*) as a function of distance between SNPs and CNVRs is shown for HOL (**A**) and JER (**B**). The types of CNVRs were defined as common deletion (0.05≤MAF; pink solid line), intermediate deletion (0.01≤MAF<0.05; pink dot dashed line), rare deletion (MAF<0.01; pink dashed line), common duplication (0.05≤MAF; blue solid line), intermediate duplication (0.01≤MAF<0.05; blue dot dashed line), rare duplication (MAF<0.01; blue dashed line)

**Figure S7. QQ plots for CNV quality control**


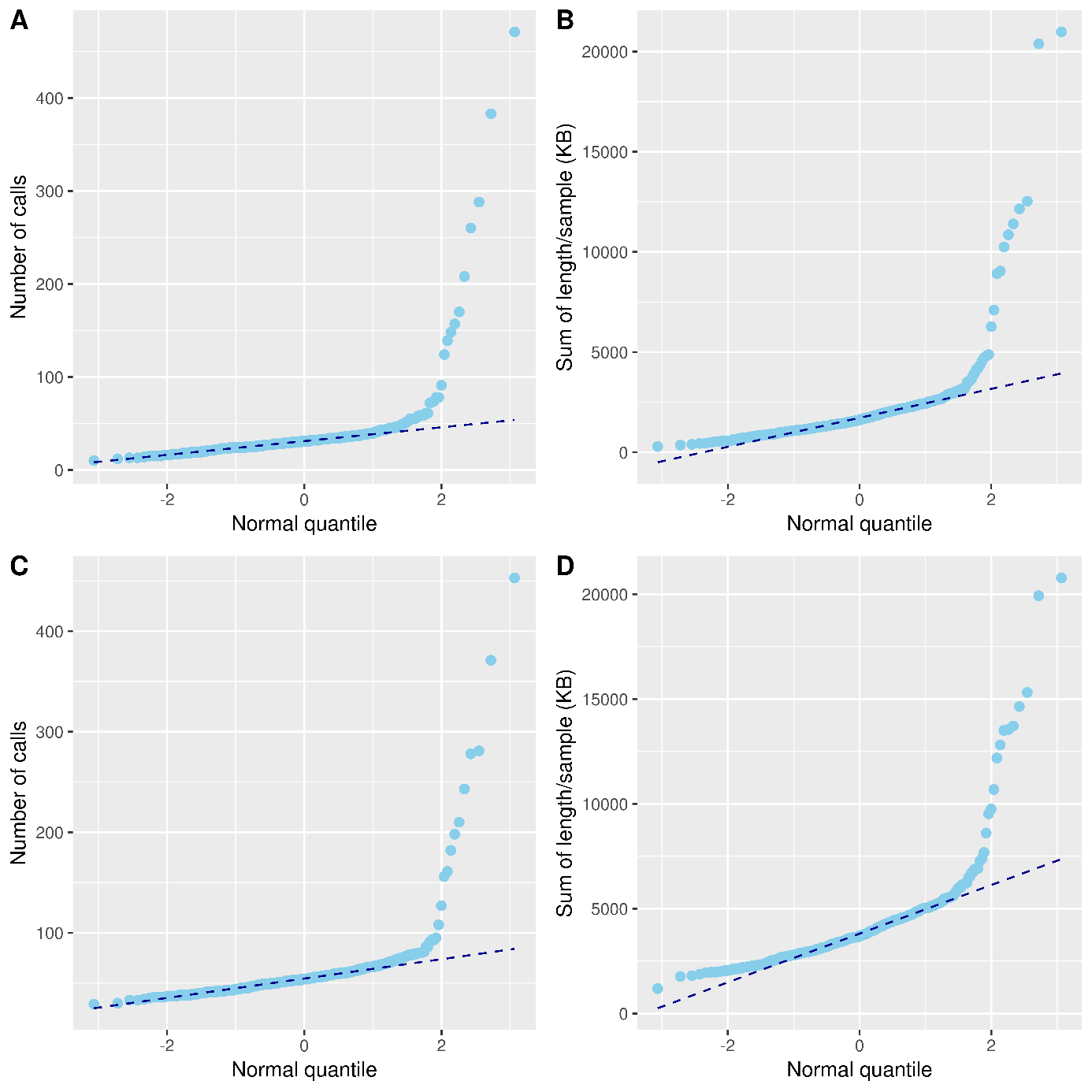


QQ plots for performing quality control on the number of CNVs per individual (Panel A for ARS-UCD1.2 and Panel C for UMD3.1) and sum of length of CNVs per individual (Panel B for ARS-UCD1.2 and Panel D for UMD3.1). The distribution was continuous until 100, and individuals with more than 100 CNVs largely deviated from the distribution (n=10). The same filter on the distribution of the total length of CNVs per individual was applied and identified outlier samples (n=11). These two filter steps identified 11 outlier individuals.
